# Deep Learning-Based Enhanced Presentation Attack Detection for Iris Recognition by Combining Features from Local and Global Regions Based on NIR Camera Sensor

**DOI:** 10.3390/s18082601

**Published:** 2018-08-08

**Authors:** Dat Tien Nguyen, Tuyen Danh Pham, Young Won Lee, Kang Ryoung Park

**Affiliations:** Division of Electronics and Electrical Engineering, Dongguk University, 30 Pildong-ro 1-gil, Jung-gu, Seoul 100-715, Korea; nguyentiendat@dongguk.edu (D.T.N.); phamdanhtuyen@gmail.com (T.D.P.); lyw941021@dongguk.edu (Y.W.L.)

**Keywords:** iris recognition, presentation attack detection, deep learning, support vector machines, NIR camera sensor

## Abstract

Iris recognition systems have been used in high-security-level applications because of their high recognition rate and the distinctiveness of iris patterns. However, as reported by recent studies, an iris recognition system can be fooled by the use of artificial iris patterns and lead to a reduction in its security level. The accuracies of previous presentation attack detection research are limited because they used only features extracted from global iris region image. To overcome this problem, we propose a new presentation attack detection method for iris recognition by combining features extracted from both local and global iris regions, using convolutional neural networks and support vector machines based on a near-infrared (NIR) light camera sensor. The detection results using each kind of image features are fused, based on two fusion methods of feature level and score level to enhance the detection ability of each kind of image features. Through extensive experiments using two popular public datasets (LivDet-Iris-2017 Warsaw and Notre Dame Contact Lens Detection 2015) and their fusion, we validate the efficiency of our proposed method by providing smaller detection errors than those produced by previous studies.

## 1. Introduction

With the development of digital technology, people are creating and managing huge amounts of information, including both public and private information, using digital systems such as computers, mobile phones, bank and government management systems, and the internet. While public information may be available for people, private information such as the information in a bank or an immigration office, assets, and other personal information is very important and should be kept private for authorized persons only. As a result, the protection of private information becomes more important in every digital system.

Traditionally, people have used two methods for this task, including knowledge-based and token-based methods [1,2]. For the knowledge-based method, each user must create a password and remember it to access a specific information resource. As the second option, the token-based method provides a key/card in which the identification information of a user is stored for accessing information resources. However, these methods incur user inconvenience in that users must remember a password for each application system or carry their key/card to access the information resources. In addition, the password and key/card can be stolen by hackers. As a result, the security level for these sources is reduced.

To overcome these limitations, biometric recognition technology has been used as an alternative [2,3,4,5,6]. This kind of recognition technology offers two advantages over the above-mentioned recognition technologies. First, biometric recognition technology uses a physical/behavioral characteristic of a human such as the face, fingerprint, or iris for recognition. As a result, users do not need to remember a password or carry a key/card. Second, as proven by a large number of studies, the biometric recognition technique offers very high recognition accuracy while reducing the potential of hacking compared to conventional methods. However, several recent studies have indicated that the biometric recognition system can be fooled by presenting artificial biometric features or attacking the recognition mechanism of recognition systems [1,7,8,9,10]. As a result, this reduces the security level of a biometric recognition system and an attack detection method is required to maintain the security level of biometric systems. While the attack detection methods for biometric features such as the face, fingerprint, and finger-vein have been studied well, the problem of iris recognition remains, especially the cross-sensor condition. Therefore, in this study, we focus on developing a high-performance presentation attack detection method for an iris recognition system (called iPAD in our study).

## 2. Related Work

The iris recognition technique has been studied for decades, and one of the first studies was performed by Daugman et al. [4]. As shown in this study, the iris recognition technique has very a high recognition rate and is reliable for real applications. Research has been performed to enhance the robustness of iris recognition system in working environments such as mobile-based iris recognition [11], iris recognition at a distance [12], non-ideal iris images [13], and noisy iris image [14]. Recently, with the development image processing technique, the deep learning-based method has been successfully applied to enhance the performance of the iris recognition system. In a study by Nguyen et al. [15], five pre-trained convolutional neural network (CNN) models (AlexNet, visual geometry group (VGG), Google Inception, ResNet, and DenseNet) were used to extract iris image features for recognition task. They showed that the deep features are superior to handcrafted image features for the iris recognition system. Lee et al. [16] used the CNN method to enhance the recognition accuracy of the iris recognition system that uses the noisy iris images as input. Liu et al. [17] and Gangwar et al. [18] the used CNN method to solve the heterogeneous iris verification/recognition problem, i.e., matching iris images across different domains such as different image resolutions or capturing conditions. To enhance the recognition accuracy, the work by Al-Waisy et al. [19] built a multi-model iris recognition that combined both left and right iris images based on CNN. The CNN method is not only used for the recognition task, but also for the iris localization task. In studies by Arsalan et al. [20,21], they used the CNN method to efficiently detect the pupil and iris boundaries that play an important role in the iris recognition system. As a result, the performance of iris recognition systems and working environment robustness is very high.

Although biometric recognition systems such as the face, fingerprint, and finger-vein have been widely used in applications, several recent studies have indicated that biometric recognition systems are vulnerable to attack threats caused by attackers presenting artificial biometric samples such as photos or 3D masks to capturing devices [1,8,9,10,22,23,24]. Similarly, researchers have found that iris recognition systems are also vulnerable to potential attack threats. To overcome this problem, several studies have been conducted to detect presentation attack images [9,25,26,27,28,29,30,31,32,33,34]. In initial studies on the iPAD problem, researchers have used handcrafted image feature extraction methods to extract image features from iris images. Then they used a classification method such as a support vector machine (SVM) to classify images into two classes of real or presentation attack based on the extracted image features [26,27,28]. Feature extractors used include the local binary pattern (LBP) and local phase quantization [26], binarized statistical image features [27], and shift-invariant descriptors [26]. In addition, eye movement information [29] and color information [30] have also been used for iPAD systems. One important limitation of the use of handcrafted image features is that the design and selection of feature extractors is mainly based on expert knowledge of researchers on the problem. As a result, the extracted image features only reflect limited aspects of the problem. Consequently, the detection performance is limited.

In recent studies, the use of handcrafted features was replaced with learning-based features or the combination of learning-based and handcrafted image features for the iPAD task [9,31]. A highlight of the learning-based feature extraction method is the application of a convolutional neural network (CNN). Silva et al. [31] used a CNN method called spoofnet to successfully classify iris images into three categories of images as textured contact lenses, soft contact lenses, and no lenses with state-of-the-art classification accuracy. In a study conducted by Menotti et al. [9], the CNN method was applied for a presentation attack detection task for three different kinds of biometric features, including fingerprint, face, and iris. A novelty of study by Menotti over the study by Silva et al. [31] is that they used two schemes of architecture optimization and filter coefficient optimization to design the CNN and therefore enhance the detection performance of the presentation attack detection system. However, the CNNs used in these studies were relatively shallow and could affect the power of the extracted image features. To overcome this problem, Nguyen et al. [25] used a deeper CNN network with 19 weight layers to learn the deep feature extractor for the iPAD system. In addition, by combining the deep and handcrafted image features extracted by the multi-level local binary pattern (MLBP) method to utilize the detection power of each kind of image feature, they enhanced the detection performance of an iPAD system further than that of previous studies using the same working dataset. 

In all of the aforementioned studies, the authors tried to extract image features using the entire detected iris region for the detection task. This approach is limited because the image features that occur during the process of artificially making a presentation attack sample can appear non-uniformly on the entire iris region. As a result, the use of the entire iris region can affect the detection performance of an iPAD system. This phenomenon suggests that the features extracted from a local iris region can be used as an alternative for features extracted from a global iris regions for iPAD. 

In a previous study [35], the authors tried to extract image features using the entire iris region presented by iris normalization step for the iPAD. We also used the inner and outer iris regions presented by iris normalization, such as in this study. This iris normalization is not the main contribution of our research, and it has been widely used in conventional iris recognition studies [4,16]. However, the performance enhancement by the previous study [35] is limited because the detailed information for iPAD along with pupil and iris boundaries, such as the discontinuity on the boundaries, are difficult to be extracted in their method. In addition, they applied the CNN method on multiple patches extracted from a normalized iris image for iPAD. An advantage of this study is that they extracted image features from local regions by dividing the input image into patches with overlapped regions. As a result, they can extract rich information in patches. However, their work is limited due to the use of many patches for iPAD that increases the processing time. In addition, the CNN network used in this study is relatively shallow, with only two convolution layers and two fully connected layers. 

To overcome these above limitations of previous studies, we propose a new iPAD method that is based on the combination of image features extracted from both local and global iris regions using a deep CNN network. In Table 1, we summarized the strengths and weaknesses of iPAD methods used in previous studies for comparison with our approach.

In the next sections of our paper, we explain the proposed method in detail as follows. Section 3 states the contributions of our study in comparison to previous studies. Section 4 provides detailed descriptions of our proposed iPAD method. Using the proposed method in Section 4, we used two popular public datasets, including LivDet-Iris-2017 Warsaw (called Warsaw-2017 in our study) and Notre Dame Contact Lens Detection 2015 (called NDCLD-2015 in our study), to evaluate the detection performance of our proposed method. The experimental results as well as a comparison with those of previous studies using the same datasets are presented in Section 5. Finally, Section 6 provides the conclusions of our work.

## 3. Contributions

In this study, we focused on enhancing the detection performance of an iPAD system by combining image features extracted from both local and global regions of iris image by the CNN method. Our study is novel in the following four aspects.

- First, to the best of our knowledge, our work is the first study that employs image features extracted from both local and global regions of iris image for an iPAD system. To overcome the limitation of previous studies which use features extracted from only local or global (entire) iris images for the detection task, we additionally extracted image features from both local and global regions of an iris image using a deep CNN network to enhance the power of the extracted image features.
- Second, we adaptively defined the local regions based on the detected boundaries of the pupil and iris so that the extracted features from these regions were robust to changes in pupil and iris sizes caused by illumination variation and distance changes between the camera and user’s eyes.
- Third, we used three kinds of input image for the detection task, including a three-channel gray-level image, a three-channel Retinex image, and a three-channel image of a fusion of the gray and Retinex image for each local and global region instead of using the gray image directly as in previous iPAD studies. Through extensive experimentation, we demonstrate the efficiency of the fusion images for the detection task.
- Fourth, we trained deep CNNs to extract deep image features for each local and global iris region image. We enhanced the detection performance by combining the features extracted from local and global regions of an iris image using two combination rules of feature level fusion and score level fusion based on SVMs. Finally, we made our trained models of CNN and SVM with all the algorithms available through [36] for access by other researchers.

## 4. Proposed Method

### 4.1. Overview of Proposed Method

**Figure 1:**
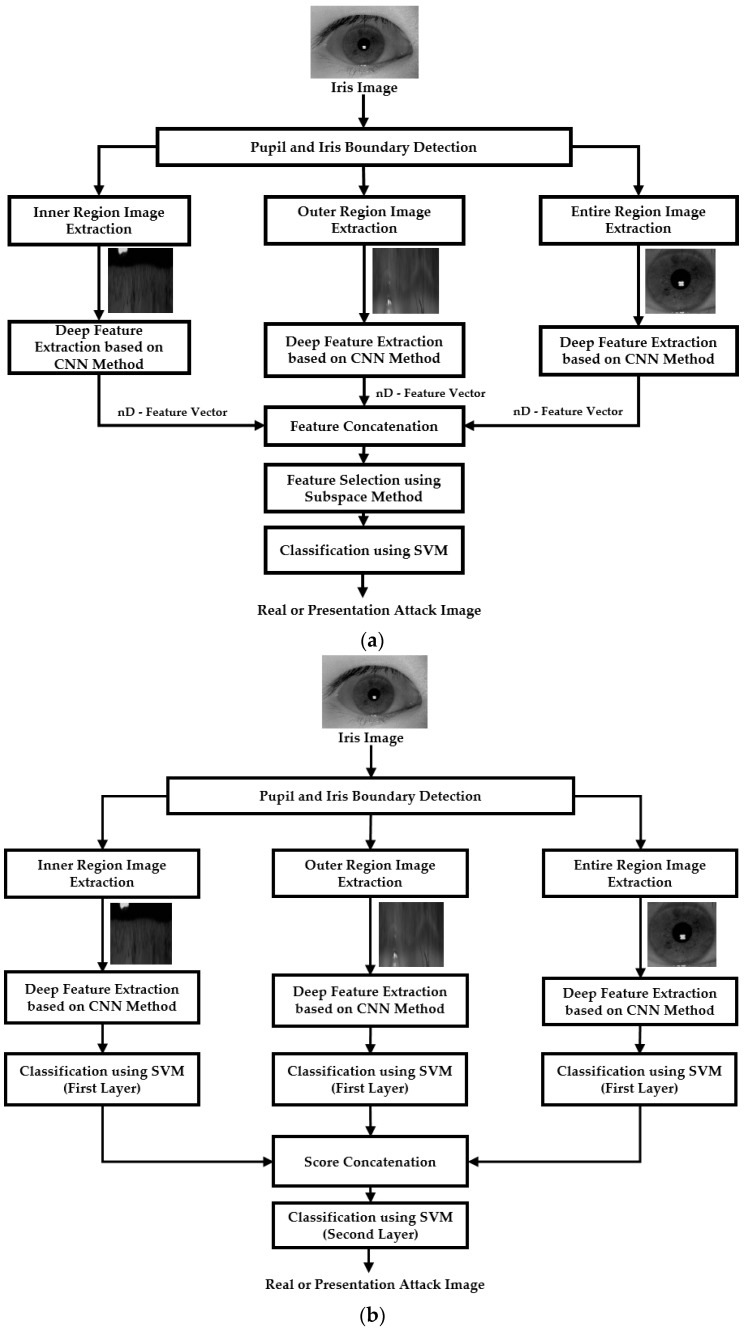
Overview flowchart of our proposed method for iPAD: (**a**) feature level fusion (“nD-Feature Vector” denotes n-dimensional feature vector), and (**b**) score level fusion.

We focused on enhancing the detection performance of an iPAD system in our study. For this purpose, we proposed a new detection framework shown in Figure 1 which utilizes the information from two local iris regions (inner and outer regions) and a global iris region. In our study, we defined the “local iris region” as an image region which covers a part of iris region in captured iris image; and the “global iris region” as the entire iris region. We used two approaches to combine the information from the local and global iris regions, including feature level fusion (Figure 1a) and score level fusion (Figure 1b). As shown in these figures, our proposed method began with a preprocessing step responsible for detecting the iris region (inner and outer iris boundaries) where an artificial iris can appear in a captured iris image. Based on the detection results of this step, we continued defining three iris regions including an inner iris region, an outer iris region, and the entire iris region for extracting the information for the detection task. The detailed explanation of these steps is given in Section 4.2.

With the two local and global iris regions, we used the CNN method to extract the image features for each region. The CNN is a very effective learning-based method for image-based classification and image feature extraction which has been successfully used for various digital signal processing applications [37,38,39,40,41,42,43,44,45,46]. As a result, we extracted three image feature vectors for the corresponding three iris regions. As the final step of our proposed method, we used the SVM method to combine the extracted image features and classify the iris images into real or presentation attack classes. For the feature-level fusion approach, the extracted image features from three iris regions were concatenated to form combined features for the iPAD. For the score-level fusion approach, we first used the SVM method to classify the three input iris region images into real and presentation attack classes. As a result, we obtained three decision scores representing the probabilities of inner, outer, and entire iris region belonging to real or presentation attack classes. Based on these decision scores, we used another SVM layer to combine the information from each local region and classify the input iris images into real or presentation attack classes as shown in Figure 1b. 

### 4.2. Iris Detection and Adaptive Definition of Inner and Outer Iris Regions

In the first step of our proposed method, we detected the boundaries of the iris region in the captured iris image. As explained in Section 4.1, our proposed method was based on the extracted image features from three different iris regions, i.e., the entire region and two local regions. Therefore, this step is important for accurately defining the iris region and its local regions. Similar to the iris recognition system, this step is necessary because the iris recognition system only uses the iris region for recognition, and an attacker can only attack the recognition system by creating an artificial iris region. As a result, the difference between a real and a presentation attack image occurs only on the iris region.

In a recent study, Cheng et al. [47] proposed a deep-learning based method for joint iris detection and presentation attack detection. However, the iPAD was done based on the roughly detected rectangular region of iris, and the detail information for iPAD along with pupil and iris boundaries are difficult to be extracted in their method. Different from this study, our iPAD uses the information from local iris regions based on pupil and iris boundaries, and more detail information for iPAD can be extracted. In addition, the iPAD is mainly used to enhance the security level of an iris recognition system. Therefore, it is usually used after an iris recognition and required to execute if an input iris image is successfully accepted as an authentic one. Therefore, the step of iris and pupil boundary detection can be shared between the iris recognition and iPAD, and our iPAD method can be adopted in conventional iris recognition system.

**Figure 2:**
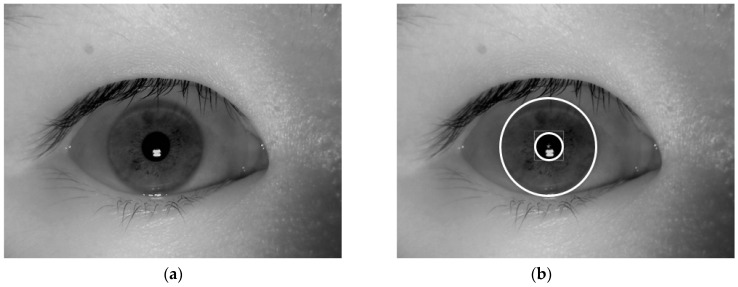
Examples of detection result of iris detection method: (**a**) a near-infrared (NIR) iris image, and (**b**) detection result of the NIR iris image in (**a**).

To detect the boundaries of the iris region of an input iris image, we used a combination of a sub-block-based template matching for rough iris detection and a circular edge detection method (CED) for fine iris boundary detection [25]. For the CED, two circular edge detectors which measure the gray difference between the inner and outer circles scanned the candidate region of the iris detected by sub-block-based template matching. The positions where the gray differences were maximum were determined as the iris and pupil regions. A detailed explanation of the detection algorithm is provided in our previous study [25]. In Figure 2, we showed an example of the detection result of the iris detection method. As shown in this figure, we efficiently detected the iris and pupil boundaries using our detection method.

**Figure 3:**
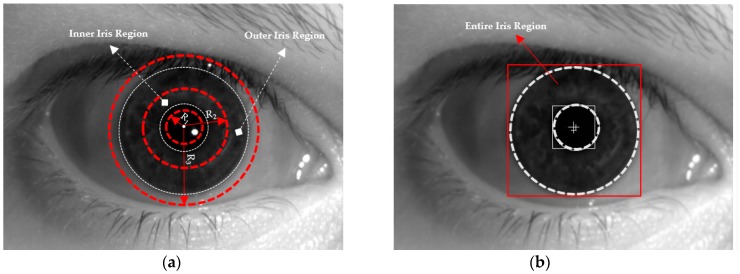
Definition of local and global iris region: (**a**) inner and outer local iris region (two donut shapes between three red circles whose radiuses of *R*_1_, *R*_2_, and *R*_3_), and (**b**) entire iris region (rectangular box).

Based on the detection results of the iris boundary detection method, we continued to define the entire iris region and two local regions for our proposed iPAD method. We obtained two center positions and the radius of the pupil and iris region. For convenience, we denoted *R_pupil_* as the radius of the pupil region and *R_iris_* as the radius of the iris region. Based on these radius results, we adaptively defined two local regions, i.e., inner and outer iris regions with radii of *R_inner_* and *R_outer_* as shown in Equations (1) and (2). In Equations (3) and (5), the optimal parameters of *α* and *β* were experimentally determined as 0.5 and 1.1, respectively. In addition, the entire iris region is defined as the largest bounding box of the detected iris region. The definition of these regions is to ensure the selected iris regions contain as much discrimination information between real and presentation attack images as possible. In Figure 3, we demonstrated the definition of iris regions used in our study.

(1) $$R_{1}\leq R_{inner}\leq R_{2}$$

(2) $$R_{2}\leq R_{outer}\leq R_{3}$$

where

(3) $$R_{1}=\alpha \cdot R_{pupil}$$

(4) $$R_{2}=\frac{R_{pupil}+R_{iris}}{2}$$

(5) $$R_{3}=\beta \cdot R_{iris}$$

**Figure 4:**
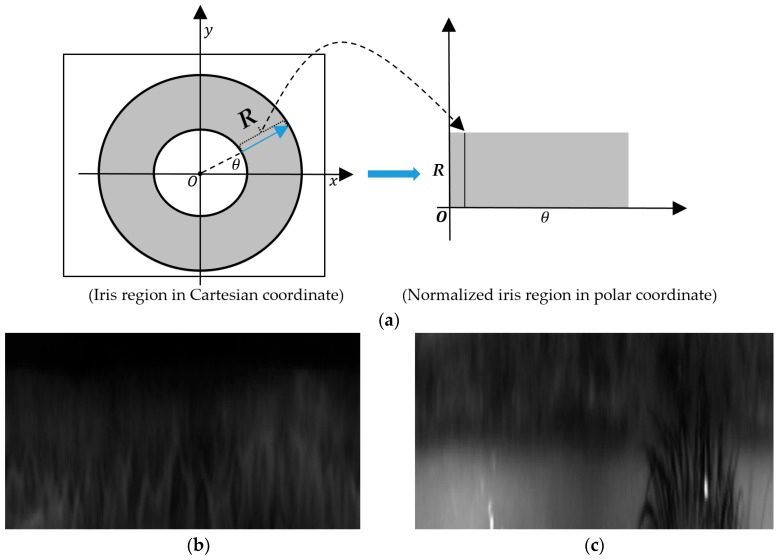
Normalization method of inner and outer iris regions: (**a**) normalization of iris region from Cartesian to polar coordinates, (**b**) normalized inner iris region of Figure 3a,c normalized outer iris region of Figure 3a.

The whole pixels inside rectangular box in red color are used as global region as shown in Figure 3b. However, the pixels between the smallest and 2nd smallest dashed circles in red color are used as inner local iris region whereas those between the 2nd smallest and largest dashed circles in red color are used as outer local iris region as shown in Figure 3a. Because the inner and outer local iris regions are donut shapes, they cannot be presented by the rectangular region, in the same manner that the entire (global) iris region in Figure 3b. Among the three selected iris regions, the inner and outer regions are defined as a circle region. Therefore, they cannot be directly used as inputs to the feature extraction method based on CNN. As a subsequent preprocessing step, we converted the inner and outer regions of the circle region to a rectangular regions as shown in Figure 4a. As shown in the region definition in Figure 3a, these inner and outer regions are defined as a donut shape. To create a rectangular image, we transform this region in Cartesian coordinate (*x*, *y*) to that in polar coordinate (*R*, *θ*) as shown in Figure 4a, and this scheme has been widely used in iris recognition researches [4,16]. In Figure 4b,c, we showed an example of the normalized iris regions in our experiment. As a result of this step, we obtained three iris region images for our iPAD algorithm, including the entire iris region image shown in Figure 3b and the two local iris region images shown in Figure 4b,c.

### 4.3. Retinex Filtering for Illumination Compensation

**Figure 5:**
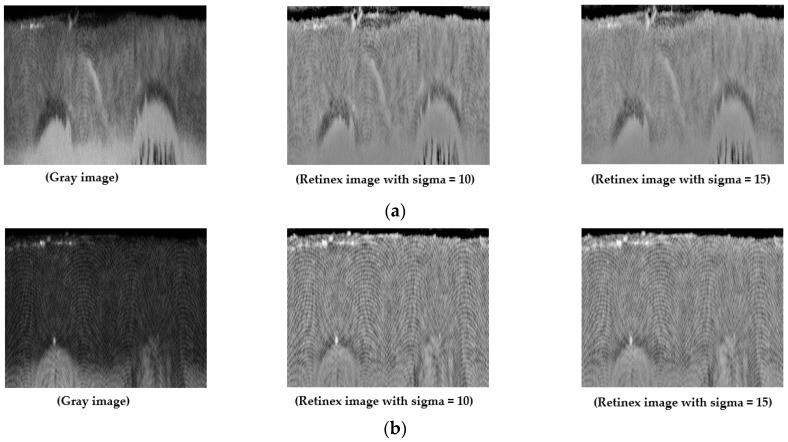
Example of result image by Retinex method: (**a**) normal-illumination gray iris image (leftmost) and its Retinex filtering results (center and rightmost), and (**b**) low-illumination gray iris image (leftmost) and its Retinex filtering results (center and rightmost).

The performance of computer vision systems is normally affected by the variation of illumination on input images. This problem also occurs in the iris recognition system because of variations in the image acquisition environment. In Figure 5, we showed two example iris images with a large difference of illumination on the leftmost side. The recognition/detection performance of a biometric system can be reduced because of the difference in illumination among images. To overcome this problem, we used the Retinex filtering technique to reduce the variation of illumination in the iris image [3]. 

In the computer vision research field, a captured image (*I(x*, *y)*) can be modeled by the multiplication of two components of an illumination component (*I_i_(x*, *y)*) and a reflection component (*r(x*, *y)*) as shown in Equation (6). While the reflection component (*r(x*, *y)*) denotes the characteristic of the texture of objects, the illumination component (*I_i_(x*, *y)*) denotes the effects of illumination sources on the captured image. Based on this assumption, the goal of the Retinex algorithm is to obtain an image that depends greatly on the reflection and that reduces the effect of illumination on the output image.

(6) $$I\left(x,y\right)=I_{i}\left(x,y\right)\times r\left(x,y\right)$$

As shown in Equation (6), in the Retinex algorithm, we tried to obtain *r*(*x*, *y*) from the captured image. Using this equation, taking the logarithm of the two sides of the equation, we obtained Equation (7). In the Retinex technique, the illumination component is assumed to be a Gaussian-blurred version of the captured image as shown in Equation (8). In this equation, *G*(*x*, *y*) is a 2-D Gaussian blur kernel with a standard deviation of σ. As a result, we obtained the reflection components as shown in Equation (10) as follows. Using Equation (10), we obtained an output image that depended more on the reflection component than on the illumination component using a suitable blur degree of the Gaussian kernel.

(7) $$\log r\left(x,y\right)=\log I\left(x,y\right)-\log I_{i}\left(x,y\right)$$

(8) $$\log I_{i}\left(x,y\right)=\log\left[I\left(x,y\right)*G\left(x,y\right)\right]$$

(9) $$G\left(x,y\right)=\frac{1}{2\pi \sigma^{2}}e^{-\frac{x^{2}+y^{2}}{2\sigma^{2}}}$$

(10) logr(x,y)=logI(x,y)−log[I(x,y)∗G(x,y)]

Using the two iris region images (gray image) on the leftmost side of Figure 5, we showed two examples of the corresponding results of the Retinex algorithm on the right side. As shown in this figure, although two input images were collected under different illumination conditions, the output images by the Retinex method had more similar balanced illumination than the input images. To evaluate the effects of illumination on the iPAD system, we measured and compared the detection performances by gray image, Retinex image, and a combination of gray and Retinex images.

### 4.4. Feature Extraction by CNN Method

As explained in Section 4.1, our proposed method used a CNN method to extract the image features for each local or global iris region. This method is an up-to-date supervised learning-based method which has received much attention and success for image classification and image feature extraction [37,38,39,40,41,42,43,44,45,46]. The success of the CNN method is based on two main operations of convolution operation responsible for the extraction of features from sources (image, voice, and text) and classification of features using a neural network (dense-connection). The convolution operation is normally associated with several other operations such as normalization and pooling. As a result, these operations make the extracted image features by CNN method robust to the image translation which normally occurs with image-based systems. With the image features extracted by the convolution operation, the CNN method uses a dense-connection (fully-connected) to learn a classifier to classify the input source into several desired classes. As proven by a variety of studies, the CNN method is suitable for various computer vision systems such as handwriting classification [48], image classification [37,38,39,40], image feature extraction [1,25,44,49], and object detection [42,43]. Inspired by the success of the CNN method, we used this method for image feature extraction in our study. 

We constructed a CNN network based on a very popular and successful network called VGG-Net-19 [38]. The detailed description of the CNN network is provided in Table 2. In detail, this network contained 19 weight layers (16 convolution and 3 fully-connected layers). Since we were investigating presentation attack detection, the output of this network contains only two possible cases of real attack or presentation attack. Therefore, the last layer of the CNN network in Table 2 contains only two neurons instead of the 1000 neurons of the original VGG-Net-19 network. With this CNN network, we performed training procedure using a training dataset to learn the filter coefficients for extracting image features and weights as a classifier to classify the extracted image features into real and presentation attack classes using a back-propagation algorithm. Finally, we used the trained CNN model to extract image features for our iPAD system. In detail, we used the features at the second fully connected layer to represent the input image. As a result, we extracted a 4096-dimensional feature vector for each iris region in our study. 

However, as reported in several previous studies [37,38], the training of CNNs is normally affected by over-fitting problem because the CNN contains a large number of weights and the training procedure needs to optimally estimate all of these weights. Fortunately, several methods have been proposed to address this problem. In our study, besides the use of a dropout layer as shown in Table 2, we used two additional methods to reduce the negative effects of over-fitting problem. First, we generalized the training dataset by using a data augmentation method [1,25,37]. By using a large generalized dataset, the network parameters can be learned efficiently because of the richer information contained in the augmented training dataset. Second, we initialized the weights of our CNN using a pre-trained VGG-Net-19 network which was successfully trained using the ImageNet dataset [38].

**Figure 6:**
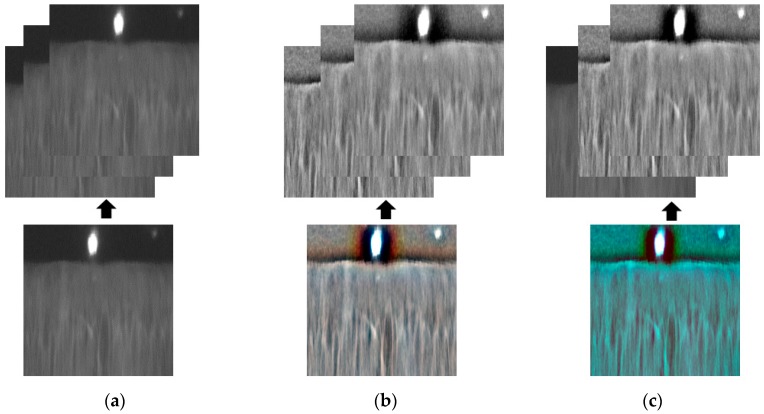
Demonstration of three-channel input image to CNN: (**a**) three-channel gray image, (**b**) three-channel Retinex image with sigma of 10, 15 and 20, and (**c**) three-channel fusion of one gray and two Retinex images with sigma of 10 and 15.

As shown in Table 2, the CNN network used in our study required a three-channel image as its input. However, the captured iris image using NIR camera sensor is normally given as a type of gray image (single-channel image). To create input images suitable for the requirement of the CNN network in Table 2, we performed a concatenation of three single-channel images as shown in Figure 6. As explained in Section 4.3, our study used the Retinex filtering method to compensate for the variation of illumination of raw iris images. To validate the efficiency of the illumination compensation method on detection accuracy, we performed experiments using three different kinds of input images as shown in Figure 6, including the use of a three-channel gray image, a three-channel Retinex image, and a three-channel image of fusion of gray and Retinex images. We show that the variation of illumination has negative effects on the detection performance and the use of Retinex method helps to reduce these effects.

### 4.5. Fusion of Detection Results by Global and Local Regions

As explained in Section 4.4, we extracted a 4096-dimensional feature vector for each local or global iris region for classification. As the final step of our proposed method, we used an SVM to classify the images into real or presentation attack images using the extracted feature vectors from local and global iris regions. As shown in Figure 1, we combined the information from all three iris regions to enhance the detection accuracy of the iPAD system. For this purpose, we invoked two combination methods, including feature level fusion and score level fusion [25,49]. As the first combination method, the feature level fusion was done by concatenating the extracted feature vectors from the three iris regions to form a new concrete feature vector as shown in Figure 1a. By concatenating the three mentioned feature vectors, the combined feature vector was supposed to contain richer discrimination information than a single local or global iris region. As a result, it was more suitable for presentation attack image detection than the use of a single feature vector. Based on this combined feature vector, we performed the classification using an SVM. The SVM is an efficient classification method based on the use of support vectors [49,50,51]. Suppose we have a training dataset that contains *n* images of two classes, and each image is represented as a *k*-dimensional feature vector. The SVM method then selects a small group from the *k*-dimensional feature vectors (called support vectors) to construct a classifier to classify the *n* images into two classes using Equation (11).

(11) $$f\left(x\right)=sign(\overset{k}{\underset{i=1}{\sum }}a_{i}y_{i}K\left(x,x_{i}\right)+b)$$

In this equation, ***x_i_*** and ***y_i_*** denote the selected support vectors and their corresponding class labels, *a_i_* and *b* are the classifier parameters obtained during the training process, and *K(**x***, ***x_i_****)* is a kernel function used to transform an input feature vector to another space (normally to a higher dimensional space) in which the classification can be easily performed. In our experiment, we used three popular kernel functions, including the linear, radial basis function (RBF), and polynomial kernel function as shown in Equations (12)–(14) [49,50].

(12) $$\mathrm{Linear}\;\mathrm{kernel}:\;K\left(x_{i},x_{j}\right)=x_{i}{}^{T}x_{j}$$

(13) $$\mathrm{Radial}\;\mathrm{basis}\;\mathrm{function}\;\mathrm{kernel}:\;K\left(x_{i},x_{j}\right)=e^{-\gamma \|x_{i}-x_{j}\|{}^{2}}$$

(14) $$\mathrm{Polynomial}\;\mathrm{kernel}:\;K\left(x_{i},x_{j}\right)={\left(\gamma x_{i}{}^{T}x_{j}+coef\right)}^{degree}$$

As the second combination method (i.e., score level fusion), we first performed the real and presentation attack image detection separately using the extracted feature vector for each iris region using an SVM. As a result, we obtained a decision score of how likely a given iris region looks like a real or presentation attack image. To combine the results of the three iris regions, the three decision scores were concatenated to form a score vector for iPAD called a score-fusion vector in our study. Finally, we used another SVM to classify the input images into real or presentation attack classes using the score-fusion vector. The flowchart of this combination method is shown in Figure 1b.

As shown in Figure 1a,b, our proposed iPAD method used the SVM method for classification instead of using the fully-connected layers of the CNN method. As proven by previous studies [25,49], this approach is effective for enhancing the detection results of a detection system. However, this approach has a limitation that the SVM must process the input feature vector in a very high dimension (4096-dimensional space with score level fusion and 12,288-dimensional space with feature level fusion). To overcome this problem, we invoked the principal component analysis (PCA) method to select a small number of efficient features for SVM instead of using the entire original features [25,52]. For this purpose, the extracted image features were first normalized using the z-score normalization method as denoted in Equation (15). In this equation, $f_{mean}$ and σ are the mean and standard deviation feature vector, respectively, obtained by using a training dataset.

(15) $$f_{norm}=\frac{f-f_{mean}}{\sigma }$$

With the normalized features, the PCA method was performed by constructing a transformation matrix W using eigenvectors corresponding to several of the largest eigenvalues of a covariance matrix constructed using the training dataset [52]. In our experiments, the optimal number of principal components with the smallest detection error was experimentally obtained. 

## 5. Experimental Results and Discussions

### 5.1. Experimental Datasets and Criteria for Detection Performance Measurement

To evaluate the performance of our proposed system and compare it with previous studies, we used two popular public datasets, including the NDCLD-2015 and Warsaw-2017 presentation attack iris dataset in our experiments. These two datasets are available through internet request and have been widely used in previous studies of iPAD systems. Although there are several other presentation attack iris datasets such as the Clarkson [34], PAVID [53], or IIITD-WVU [34], these were not available through internet request. The two datasets we chose were also used in previous iPAD study [34] for LivDet-2017-Iris competition. In Table 3, we showed the detailed description of these two datasets regarding their sizes and image acquisition methods. As shown in this table, the Warsaw-2017 dataset is larger and contains a total of 12,013 images (5168 real and 6845 presentation attack). The presentation attack images were collected by recapturing several printed iris images on paper. Because of this collection method, the presentation attack iris images in the Warsaw-2017 dataset contain much noise and/or broken textures. In contrast to the Warsaw-2017 dataset, the NDCLD-2015 dataset simulates another attack method based on the contact lens for attacking the iris recognition system. As reported by previous studies, this attack method can produce an iris image more similar to that of a real image than the attack method used for collecting images in the Warsaw-2017 dataset. As shown in Table 3, the NDCLD-2015 dataset contains a total of 7300 images in which 4785 are real images and the remainder are presentation attack images. Using these two datasets, we measured the detection performance and compared it with those of previous studies to validate the efficiency of our proposed method.

To measure the performance of an iPAD system, we refer to the ISO/IEC-30107 standard [54,55]. In this standard, two error measurement criteria are used, including the attack presentation classification error rate (APCER) which represents the proportion of presentation attack images incorrectly classified as bona fide (real) presentations using the detection system and the bona fide presentation classification error rate (BPCER) which represents the proportion of bona fide presentation images incorrectly classified as presentation attack images. These two error measurements have trade-off properties. Therefore, the average value of these two error measurements, called the average classification error rate (ACER), is normally used to measure the performance of a detection system as shown in Equation (16). Since the APCER and BPCER are both classification error measurements, the ACER also indicates the detection error of a detection system. As a result, a small ACER value indicates a better detection system performance.

(16) $$\mathrm{ACER}=\frac{APCER+BPCER}{2}$$

In our experiments, we measured the performance of our proposed method using all of these measurement criteria according to various numbers of principal components.

### 5.2. Performance Evaluation of Individual Attack Method

As explained in Section 5.1, we used two datasets (NDCLD-2015 and Warsaw-2017) in our experiments to evaluate the detection of our proposed method. In this section, we measured the detection performance of our proposed method using each individual dataset to investigate the detection performance regarding each type of attack method, i.e., printed sample on paper and contact lens.

#### 5.2.1. Detection Performance of Attack Method Based on Iris Image Printed on Paper

In the first experiments, we evaluated the performance of our proposed method for detecting a printed sample of an iris image. For this purpose, we used the Warsaw-2017 dataset. As explained in Section 5.1, the Warsaw-2017 dataset contains a total of 12,013 images. Among these images, 4513 images were predefined as training images, and the other 4510 images were predefined as testing images by the author of the database. This predefinition helps to fairly compare the detection accuracy among iPAD studies. To reduce the effect of over-fitting problem caused by the CNN method, we enlarged (generalized) the size of the training dataset by artificially making augmented images from each original image. For the entire iris image, we used the shifting and cropping method. For the two local regions (inner and outer), we created artificial images by applying a small error to the detection results of the iris detection algorithm. A detailed description of the training and testing datasets in this experiment is provided in Table 4. We used 51,681 images for training and 4510 images for testing. The size of the testing dataset remained as predefined one to fairly compare our detection performance with those of previous studies.

Using the augmented dataset presented in Table 4, we performed experiments for three kinds of input images (using a three-channel gray image, a three-channel Retinex image, and a three-channel fusion of gray and Retinex images) and five detection approaches including detection methods using only the inner iris region; only the outer iris region; using only the entire iris region; using the feature level fusion of inner, outer, and entire iris region; and using the score level fusion of inner, outer, and entire iris region. 

In Table 5a, we measured the accuracies by the method which directly used CNN to train the iPAD and produce the presentation attack (PA) scores whereas our detection results are shown in Table 5b. As shown in these tables, our method outperforms that directly used CNN.

**Figure 7:**
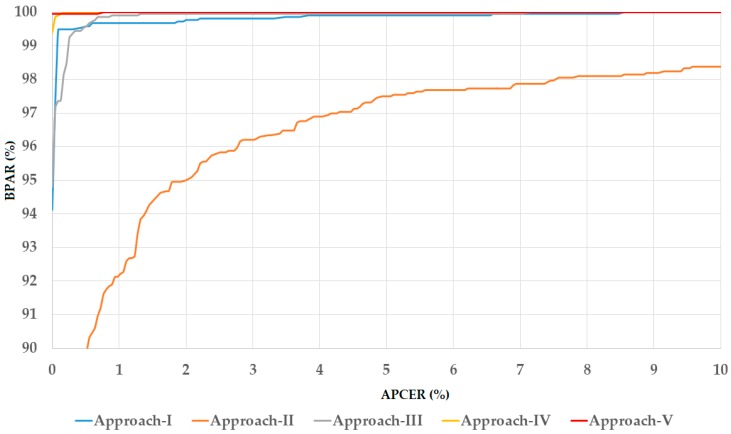
Detection error trade-off (DET) curves of iPAD systems according to best detection accuracy presented in Table 5b using three-channel images of fusion of gray and Retinex images for iPAD.

In the upper part of Table 5b, we showed the detection error of the five detection approaches using the test-known dataset. As shown in these results, we obtained perfect detection results using our proposed method (using feature or score level fusion approaches) by producing an error rate (ACER) of 0.000%. These results were better than those of using only inner or outer iris regions and equal to that of using entire iris region. This detection result was obtained because the test-known dataset was collected using the same camera and acquisition procedure as the training dataset. Therefore, the test-known dataset had similar characteristics to those of the training dataset. However, the detection errors increased using the test-unknown dataset as shown in the lower part of Table 5b. Again, our proposed approach (using feature or score level fusion of local and global iris regions) outperformed the other approaches that used a single iris region for detection task. Using the three-channel gray images, we obtained the best detection accuracy of 0.153% and 0.087% using our approaches. These errors were smaller than those produced by using single local or global iris region which produced errors of 0.268%, 0.713%, and 0.589%, respectively. Similarly, we obtained the best detection accuracy with an ACER of 0.222% using the three-channel Retinex images and 0.023% using the three-channel fusion of gray and Retinex images which were much smaller than the other detection errors in Table 5b produced by other approaches using the test-unknown dataset. Both of these smallest detection errors were obtained using our proposed approach based on score level fusion. The detection errors in the experiments with the test-unknown dataset were higher than those using the test-known dataset because of the difference in image characteristics between the test-known and test-unknown dataset. Since the test-unknown dataset was acquired using a different camera than that of the test-known and training dataset, the resultant image characteristics of the test-known and test-unknown were very different. However, as shown in our experimental results, the detection results produced by the test-unknown dataset were also very small and close to zero using our approach. Based on this detection error, we demonstrated that our proposed approach produced very high detection performance using the Warsaw-2017 dataset, and the fusion of gray and Retinex images was sufficient for the iPAD. For demonstration purposes, we showed the detection error trade-off (DET) curve of the best detection results presented in Table 5b which is the result of our proposed approach using score level fusion and the fusion of gray and Retinex images for iPAD in Figure 7. In this curve, we drew the change of APCER according to the bona fide presentation acceptance rate (BPAR) measured by (100—BPCER) (%). The DET curves for these experiments using test-known dataset are not shown because we obtained a perfect detection performance for the test-known dataset. As shown in this figure, the proposed method (the red line according to the proposed method based on score level fusion) outperformed the other iPAD methods.

#### 5.2.2. Detection Performance of Attack Method Based on Use of Contact Lens Using the LivDet-Iris-2017 Division Method

As the second experiment in our study, we evaluated the detection performance using the NDCLD-2015 dataset to verify the detection performance of our proposed method with the second kind of attack method based on the use of a contact lens. As explained in Section 5.1, the NDCLD-2015 dataset has been used in the LivDet-Iris-2017 competition for liveness iris detection. In this competition, the NDCLD-2015 dataset was used by selecting 1200 images (600 real and 600 presentation attack images) as the training dataset and the 1800 images (900 real and 900 presentation attack images) as the testing dataset. Two testing datasets were considered, including a test-known dataset whose images were collected by using the same contact lens manufacturers with the training dataset and a test-unknown dataset whose images were collected using contact lenses of different manufacturers with the training dataset. With the purpose of measuring the detection performance of our proposed method for iPAD as well as comparing it with previous studies, we first performed similar experiments with the LivDet-Iris-2017 competition. However, the detailed information of which images were selected as the training and testing datasets was not available through internet request. Therefore, we performed our experiments by randomly selecting images for training and testing datasets using the same criteria as the LivDet-Iris-2017 competition. To ensure the convergence of the detection accuracy, we performed the experiment twice (a two-fold cross-validation) to measure the detection accuracy. As a result, the final detection accuracy was measured as the average of the two detection results of the two folds. We also performed a data augmentation procedure to generalize the training dataset. A detailed description of the datasets used in this experiment is provided in Table 6. As shown in this table, we used 58,800 images for training and 1800 images for each test-known and test-unknown dataset.

Similar to our experiments in Section 5.2.1, we performed experiments using the dataset in Table 6 with three kinds of input image and five detection approaches (the detection methods using single local or global iris region and the fusion of the three regions), and the detailed detection accuracies are shown in Table 7a,b. In Table 7a, we measured the accuracies by the method which directly used CNN to train the iPAD and produce the PA scores whereas our detection results are shown in Table 7b. As shown in these tables, our method outperforms that directly used CNN.

First, we showed the detection accuracies (APCER, BPCER, and ACER) using the test-known dataset in the upper part of Table 7b. We obtained a perfect detection using our proposed method (using feature or score level fusion approaches) for all the cases using either a three-channel gray image, a three-channel Retinex image, or a three-channel fusion of gray and Retinex images. Compared to the detection errors produced by using the approaches that use a single region image (only inner, only outer, or only entire iris region) for the detection task, the detection errors of our proposed method were lower as shown in the upper part of Table 7b. This situation is quite similar to our experiments with the test-known dataset of the Warsaw-2017 dataset because the images in training and test-known datasets were acquired using the same contact lens manufacturers. As a result, the presentation attack images in these two datasets exhibited similar characteristics.

**Figure 8:**
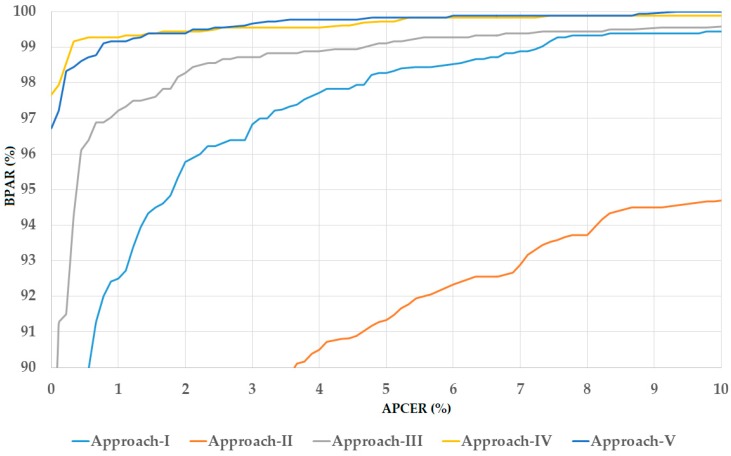
DET curves of iPAD systems according to best detection accuracy in Table 7b using the three-channel images of fusion of gray and Retinex images for iPAD.

In the lower part of Table 7b, we showed the experimental results using the test-unknown dataset. Using the three-channel gray images, we obtained the best detection accuracy of 1.722% using our proposed approach with the feature level fusion. This detection error was smaller than that using single iris region images and score level fusion. Similarly, we obtained the smallest detection error of 0.611% using the three-channel Retinex images and an error of 0.583% using the three-channel image of fusion of gray and Retinex images with feature level fusion of three iris region images. Since we were working with the test-unknown dataset, these detection errors were higher than those produced by the test-known dataset in the upper part of Table 7b. However, these detection errors were much smaller than those produced by previous studies and are compared in detail in Section 6. Again, we obtained the smallest detection error using our proposed method with the three-channel fusion of gray and Retinex images. This result demonstrates that the variation in illumination has a strong effect on the detection system and the use of Retinex technique can help to enhance the detection accuracy. For demonstration purposes, we showed the DET curves of experiments using our proposed method with three-channel fusion images in Figure 8. We again only drew the DET curves for the use of test-unknown dataset because we obtained a perfect detection performance using the test-known dataset. This figure again clearly demonstrates the higher performance of our proposed method by presenting the two curves of feature level fusion and score level fusion at a higher position than those of the other approaches.

#### 5.2.3. Detection Performance of Attack Method Based on Use of Contact Lens Using Our Division Method

The division of images into training and testing datasets mentioned in Section 5.2.2 was used in the LivDet-Iris-2017 competition. Performing experiments with this division method helped us to evaluate the performance of our proposed method in the same framework as previous studies. However, this division method has two limitations. First, it considers the iris images of contact lenses (even transparent contact lenses) as presentation attack images. Many people use transparent contact lenses to protect their eyes or compensate for eye diseases such as myopia or hyperopia. For this reason, transparent contact lenses should be accepted for iris recognition system use in their daily life or work. However, using the above criteria, those wearing transparent contact lenses may be regarded as attackers. Second, the LivDet-Iris-2017 competition used only 4800 images of the NDCLD-2015 dataset for detection (1200 images for training and 1800 images for each test-known and test-unknown dataset). Therefore, 2500 images were not used. However, the use of a large number for training and testing data is usually effective for enhancing and correctly evaluating system performance. Because of these two problems associated with this division meth, we performed further experiments using our proposed new division method.

In the first proposed division method, we suggest accepting the iris image of the use of a transparent contact lens as a real image ones. Based on this new criteria, we randomly selected new training and testing datasets that were the same size as in our experiment in Section 5.2.2 which had 1200 images for training and 1800 images for each test-known and test-unknown dataset. With these new datasets, we performed experiments similar to those in Section 5.2.2, and the experimental results are given in Table 8a,b using two-fold cross-validation. In Table 8a, we measured the accuracies by the method which directly used CNN to train the iPAD and produce the PA scores whereas our detection results are shown in Table 8b. As shown in these tables, our method outperforms that directly used CNN. 

As shown in Table 8b, we again obtained a detection error of 0.000% using the test-known dataset using our proposed method (using either feature level fusion or score level fusion approach) and either gray image, Retinex image, or fusion of the two. Using the test-unknown dataset, we obtained the smallest detection errors of 1.500%, 0.500%, and 0.944% using our proposed method with feature level fusion approach using a three-channel gray image, a three-channel Retinex image, and a three-channel image of fusion of the two, respectively. The best detection accuracy with an ACER of 0.50% was obtained using our proposed method and a Retinex image. This detection error was smaller than the detection error of 0.583% produced in our experiment in Section 5.2.2 using the LivDet-Iris-2017 division method because we considered the iris images of a transparent contact lens to be real images. As a result, it increased the discrimination between real and presentation attack classes because the iris images with and without a transparent contact lens exhibit a real iris pattern that is different from artificial iris patterns. 

As the second proposed division method, we used the entire NDCLD-2015 dataset for our experiment and divided it into training and testing datasets without considering the test-known and test-unknown data. This division method has two meanings. First, we use all the data for the detection task to enhance and correctly evaluate the system performance because of the use of a larger dataset. Second, we train the detection model using a training dataset with a larger variation of image data by fusing test-known and test-unknown data. Based on these criteria, we divided the entire NDCLD-2015 dataset into training and testing datasets by which half of the data were assigned as training data, and the other half as testing data. We repeated our experiments twice to perform a two-fold cross-validation procedure by exchanging the training and testing dataset of the first fold in the second fold. Consequently, we obtained two working datasets (1st Fold and 2nd Fold datasets) as shown in Table 9. The final experimental results were measured by taking the average of the two detection accuracies of the two folds and are shown in Table 10a,b. In Table 10a, we measured the accuracies by the method which directly used CNN to train the iPAD and produce the PA scores whereas our detection results are shown in Table 10b. As shown in these tables, our method outperforms that directly used CNN.

As shown in Table 10b, using our proposed method with the feature level fusion approach and a three-channel gray image, we obtained the best detection accuracy of ACER of 1.152%. This detection error was further reduced to 0.959% using a three-channel Retinex image and to 0.965% using a three-channel fusion image of the gray and Retinex images. These detection errors were smaller than those produced by other approaches, especially those using only one local region for detection which produced much larger errors than our best detection performance. In addition, the best detection using the entire iris region image was 1.337% using a three-channel fusion of gray and Retinex images. This detection error was higher than our smallest detection error of 0.959%. Based on this result, we confirmed that our proposed detection method based on both local and global iris regions was effective at enhancing detection accuracy and outperformed the use of only the entire global iris region image.

### 5.3. Performance Evaluation of Combined Datasets for Considering General Attack Method

As explained in Section 5.1 and Section 5.2, the Warsaw-2017 and NDCLD-2015 datasets simulate two different attack scenarios on iris recognition systems, including using the printed-paper iris sample and using contact lenses. In Section 5.2, we showed the detection performance of our approaches for each individual attack method. However, attackers may use one of the various possible attack methods to attack iris recognition systems. Consequently, if an iPAD system only considers a limited attack method, the attacker can easily fool the recognition system by using a different attack method. Therefore, it is a natural requirement that an iPAD system should be robust to various attack methods. One solution to this problem is to train an iPAD system using a large dataset that contains various attack methods. To validate the detection performance of our proposed method with various attack methods, we performed further experiments with a new dataset formed by fusing the two separate datasets in Section 5.2. For this purpose, we fused the training and testing datasets of the Warsaw-2017 and NDCLD-2015 datasets to form the new dataset shown in Table 11. The new training dataset contained a combination of 51,681 images from the Warsaw-2017 dataset and 58,800 images from the NDCLD-2015 dataset for a total of 110,481 images. Similarly, the two testing datasets (test-known and test-unknown) contained a total of 4790 and 6310 images, respectively. We performed experiments using this new dataset, and the experimental results are provided in Table 12a,b. In Table 12a, we measured the accuracies by the method which directly used CNN to train the iPAD and produce the PA scores whereas our detection results are shown in Table 12b. As shown in these tables, our method outperforms that directly used CNN.

**Figure 9:**
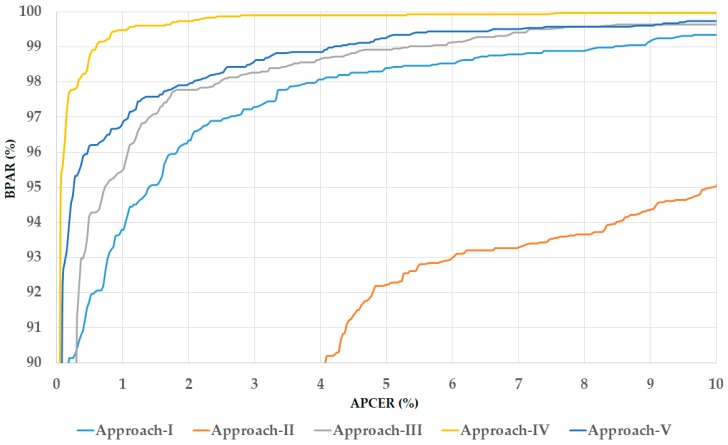
DET curves of iPAD systems according to best detection accuracy presented in Table 12b using three-channel images of fusion of gray and Retinex images for iPAD.

As shown in Table 12b, our proposed method achieved perfect detection accuracy (ACER of 0.000%) on the test-known dataset using either the feature level fusion or the score level fusion approach. This result indicates that the test-known dataset is easy to detect because of the similar characteristics with the training dataset and implies that the proposed method can obtain good detection results if we can simulate all possible attack methods in the training data. Using the test-unknown dataset, we obtained the best detection errors of 1.334%, 1.156%, and 0.709% using a three-channel gray image, a three-channel Retinex image, and a three-channel fusion of gray and Retinex images, respectively. The best detection error was approximately 0.709% obtained using our proposed method and three-channel image of fusion of gray and Retinex images. In addition, this best detection accuracy was much smaller than those produced by the use of single-region iris images as shown in Table 12b. This detection result again confirms that our proposed method with fusion images is efficient for iPAD in general. In Figure 9, we showed the DET curves of the detection systems using the three-channel image of fusion of gray and Retinex images using the test-unknown dataset. As shown in this figure, the proposed method with the feature level fusion approach outperformed the other detection approaches.

For the next experiment, we measured the processing time of the proposed method using a desktop computer with an Intel Core i7 CPU (Intel Corporation, Santa Clara, CA, USA) (3.4 GHz; 64 GB of RAM memory). For running the CNN model, we used a TitanX graphics processing unit (GPU) card [56]. As demonstrated in Table 13, proposed method requires about 84.9 milliseconds (ms) to process an input iris image. It indicates that our method can operate at the speed of about 11.77 (1000/84.9) frames per second (fps). As shown in this table, the pupil and iris boundary detection and deep feature extraction by CNN method occupies the largest processing time in our method. However, the computation cost of the pupil and iris boundary detection is shared by the iris recognition system because the iPAD system is usually used with the iris recognition. Therefore, we can think that only the proposed iPAD method can operate at a higher speed of 62.40212 (84.90212 − 22.5) ms per image, or about 16 fps.

### 5.4. Comparative Experiments with Previous Methods and Discussions

The two datasets used in the above experiments (Warsaw-2017 and NDCLD-2015) have been used for evaluating the detection performance of the iPAD method in previous studies [25,34]. In these studies, several proposed detection methods used these datasets to measure the detection accuracy. To validate the detection performance of our proposed method, we further performed a comparison of our detection performance with those of previous studies in Table 14. In this table, the final detection accuracy was measured by taking the weighted average of the test-known and test-unknown datasets according to the number of real and presentation attack images in each dataset.

First, we compared the detection accuracy of our proposed method with those of previous studies using the Warsaw-2017 dataset. In a study conducted by Yambay et al. [34], three detection methods were proposed, including the CASIA, Anon1, and UNINA methods. Using the Warsaw-2017 dataset, they reported detection accuracies (ACERs) of 6.00%, 5.81%, and 7.41%, respectively. In a recent study by Nguyen et al. [25], the authors combined the image features extracted by the CNN method and MLBP method and used an SVM for classification. As a result, they reduced the detection error (ACER) to 0.263%, 0.224%, 0.142%, and 0.016% using the CNN method, the MLBP method, a combination of CNN and MLBP using feature level fusion, and a combination of CNN and MLBP using score level fusion, respectively. As shown in Table 14, our proposed method produced a detection error of 0.016%, which is the same as the smallest detection error reported by Nguyen et al. [25] and much smaller than those reported by other studies.

Similar to the first comparison, we compared the detection performance of our proposed method with those using the NDCLD-2015 dataset, and the results are presented on the right side of Table 14. As we explained in above part of this section, the NDCLD-20105 dataset has been used in the LivDet-Iris-2017 competition. Although the information of the division of images into training and testing datasets was not available to us, we performed experiments by randomly selecting images for the training and testing datasets twice using the same criteria as the LivDet-Iris-2017 competition. Therefore, we believe that the comparison between detection performance of our proposed method and the study by Yambay et al. [34] is balanced. As shown in this table, the smallest detection error produced by previous studies was 2.098%, which was reported by Nguyen et al. [25] using a combination of CNN and MLBP features based on the feature level fusion approach. Compared to this detection error, our proposed method produced a much smaller detection error with an ACER of 0.292%. These detection results show that our proposed method outperformed previous studies and it is efficient for an iPAD.

As shown in our experimental results in the above sections, we obtained perfect detection accuracy using the test-known dataset, while we obtained much smaller detection errors than those of previous studies using the test-unknown dataset. This result is caused by the fact that the test-known dataset has similar characteristics with the training dataset because of the use of the same capturing device/presentation attack sample manufacturer and similar capturing procedure. However, the test-unknown data were collected using an unknown procedure (capturing device, environment, and manufacturer) with the training data. As a result, the distribution of real and presentation attack data in the test-unknown data was different from that in the training data and increased the detection error when using the test-unknown data.

Although we obtained a detection error on test-unknown data higher than that of the test-known data, the detection error of test-unknown data was much smaller than that produced by previous studies. As a result, the final detection error (the weighted average of test-known and test-unknown data) was much smaller than those reported in previous studies as shown in Table 14. This result confirms that our proposed method outperformed previous studies and is efficient for enhancing the security level of iris recognition systems.

The detection framework in our method is based on the idea of deep feature extraction by CNN, feature selection by PCA, and classification by SVM that is similar to the work by Nanni et al. [58]. However, this is not the main contribution of our research. As a new contribution, we propose the method of combining information extracted from both local and global iris regions to enhance the detection performance of iPAD system as stated in Section 4 and Section 5. Therefore, we can think that our method has similar performance with the work by Nanni et al. [58] when applying on only the entire iris region. However, it showed better detection performance than the work by Nanni et al. [58] while combining the information from both local and global iris region as shown in our experimental results in Table 5b, Table 7b, Table 8b, Table 10b and Table 12b.

In our study, we used an iris detection method based on a combination of sub-block-based template matching and CED. The sub-block-based method is used to find the rough position of pupil region, and the CED method can be efficiently applied to find the accurate pupil and iris boundaries. The detection errors of CED method has been measured based on the ground-truth centers and radii of iris and pupil which were manually obtained in our previous study [59]. As shown in this study, the detection errors were measured in two databases of CASIA iris open dataset and the self-collected dataset captured by mobile phone camera. Because the second dataset was collected both in indoor and outdoor, it includes various factors of uncontrolled environments. As shown in this study, the detection errors of CED method in iris center was from 3.47 to 4.83 pixels whereas those in pupil center was from 1.75 to 2.44 pixels. The detection errors of CED method in iris radius was from 2.47 to 3.13 pixels whereas those in pupil radius was from 2.3 to 2.45 pixels. Therefore, we can regard that these errors are already reflected to the results of our iPAD method. However, the detection method can be failed with the severely uncontrolled iris image due to the negative effects of capturing environment as explained in [59]. Our research is for iPAD to enhance the security of iris recognition system. Therefore, an iPAD system is usually invoked when an iris recognition system successfully recognizes an input iris image as an authentic one. As a result, if the iris detection method is failed or incorrect iris region is located by this method, the consequent recognition result by iris recognition system is also failed. For this case, our iPAD method is not performed. Therefore, the cases of detection failure or incorrect detection by the iris detection method do not affect the performance of iPAD.

## 6. Conclusions

We investigated the presentation attack image detection ability of local and global regions of the iris images and consequently enhanced the detection performance of iPAD method by combining the detection results of these regions using a fusion method. Using the Warsaw-2017 and NDCLD-2015 public datasets, we showed that the proposed method outperformed previous studies for iPAD problem. In detail, the local regions (inner and outer) can be used to extract texture features caused by the non-uniform distribution of texture features on the iris region. Using the two local regions and the entire iris region, we extracted image features using the CNN method. Finally, by combining image features extracted from both the local and global regions of an iris image, i.e., inner and outer local regions and the global region, we efficiently enhanced the detection accuracy compared to that of previous studies. In addition, we investigated the detection performance of the proposed method using three kinds of input images, including the use of three-channel gray images, three-channel Retinex images, and the three-channel images of a fusion of gray and Retinex images. As shown in our experimental results, the fusion of gray and Retinex images produced the smallest detection error. 

Although the CNN network used in our study is very deep with 19 weights layers, it is possible to use and combine different CNN networks for enhancing the performance of iPAD. In addition, we plan to investigate the effects of the depth of CNN network on the detection performance of iPAD system using shallower or deeper CNN architecture.

## Figures and Tables

**Table 1:** Summary of previous studies on iPAD compared to our proposed method.

Category	Method	Strength	Weakness
Using image features extracted from entire (global) iris region image	Uses handcrafted image features extracted from entire iris region image [26,27,28,29,30]	- Easy to implement - Feature extractors are designed by experts	Detection accuracy is fair because of predesigned image feature extraction method
	Uses learning-based method, i.e., CNN method [9,31,34]	Extracts efficient image features by a learning-based method using a large amount of training samples	- Only captures information extracted from global (entire) iris image for detection problem - Processing time for both training and testing steps is longer than that using handcrafted image features.
	Uses combination of deep and handcrafted-image features [25]	Enhances the detection performance by using both handcrafted and deep image features	- Only captures image features from global iris image for detection problem - More sophisticated than the use of only deep or only handcrafted image features.
Using image features extracted from multiple local patches of normalized iris image	- Extract overlapped local patches of iris region for classification. - Using CNN method to classify patches into real or presentation attack class [35]	- Extracts rich information from overlapped image patches. - Utilizes the learning-based method i.e., CNN, for feature extraction and classification.	- Takes long processing times because of using multiple patches. - CNN network is relatively shallow. - Does not consider the detail information along with pupil and iris boundaries
Combining features extracted from both local and global iris regions for detection task (Proposed method)	- Extracts image features from inner and outer local regions of iris image in polar coordinate system using CNN method - Extracts image features from global (entire) iris region from Cartesian coordinates - Combines detection results by features extracted from local and global iris regions using fusion rule	- Captures information from both local and global regions of image for detection task - Produces higher detection accuracy than the use of only image features extracted from global iris region, especially with the cross-sensor or cross-artificial template manufacturer condition	Processing time is longer than when using only image features extracted from global iris region

**Table 2:** Description of convolutional neural network (CNN) architecture used in our iPAD study.

Operation Layer		Number of Filters	Size of Each Filter	Stride Value	Padding Value	Size of Output Image
**Input image**		-	-	-	-	224 × 224 × 3
**Convolution Layer (two times)**	Convolution	64	3 × 3 × 3	1 × 1	1 × 1	224 × 224 × 64
	ReLU	-	-	-	-	224 × 224 × 64
**Pooling Layer**	Max pooling	1	2 × 2	2 × 2	0	112 × 112 × 64
**Convolution Layer (two times)**	Convolution	128	3 × 3 × 64	1 × 1	1 × 1	112 × 112 × 128
	ReLU	-	-	-	-	112 × 112 × 128
**Pooling Layer**	Max pooling	1	2 × 2	2 × 2	0	56 × 56 × 128
**Convolution Layer (four times)**	Convolution	256	3 × 3 × 128	1 × 1	1 × 1	56 × 56 × 256
	ReLU	-	-	-	-	56 × 56 × 256
**Pooling Layer**	Max pooling	1	2 × 2	2 × 2	0	28 × 28 × 256
**Convolution Layer (four times)**	Convolution	512	3 × 3 × 256	1 × 1	1 × 1	28 × 28 × 512
	ReLU	-	-	-	-	28 × 28 × 512
**Pooling Layer**	Max pooling	1	2 × 2	2 × 2	0	14 × 14 × 512
**Convolution Layer (four times)**	Convolution	512	3 × 3 × 512	1 × 1	1 × 1	14 × 14 × 512
	ReLU	-	-	-	-	14 × 14 × 512
**Pooling Layer**	Max pooling	1	2 × 2	2 × 2	0	7 × 7 × 512
**Inner Product Layer**	Fully connected	-	-	-	-	4096
	ReLU	-	-	-	-	4096
**Dropout Layer**	Dropout (dropout = 0.5)	-	-	-	-	4096
**Inner Product Layer**	Fully connected	-	-	-	-	4096
	ReLU	-	-	-	-	4096
**Dropout Layer**	Dropout (dropout = 0.5)	-	-	-	-	4096
**Inner Product Layer**	Fully connected	-	-	-	-	2
**Softmax Layer**	Softmax	-	-	-	-	2
**Classification Layer**	Classification	-	-	-	-	2 (Real/Presentation Attack)

**Table 3:** Description of Warsaw-2017 and NDCLD-2015 datasets.

Dataset	Number of Real Images	Number of Attack Images	Total	Image Data Collection Method
Warsaw-2017	5168	6845	12,013	Recaptured printed iris patterns on paper
NDCLD-2015	4875	2425	7300	Recaptured printed iris patterns on contact lens

**Table 4:** Description of Warsaw-2017 dataset in our experiment (with augmentation of training dataset).

Dataset	Training Dataset			Testing Dataset					
	Real Image	Attack Image	Total	Test-Known Dataset			Test-Unknown Dataset		
				Real Image	Attack Image	Total	Real Image	Attack Image	Total
Original dataset	1844	2669	4513	974	2016	2990	2350	2160	4510
Augmented dataset	27,660 (1844 × 15)	24,021 (2669 × 9)	51,681	974	2016	2990	2350	2160	4510

**Table 5:** (a) Detection errors (attack presentation classification error rate (APCER), bona fide presentation classification error rate (BPCER), and average classification error rate (ACER)) of iPAD based on CNN method for classification using Warsaw-2017 dataset and three different kinds of input image (unit: %); (b) Detection errors (APCER, BPCER, and ACER) of iPAD based on SVM method for classification using Warsaw-2017 dataset and three different kinds of input image (unit: %).

Test Dataset	Approach	Using Three-Channel Gray Images			Using Three-Channel Retinex Images			Using Three-Channel Fusion of Gray and Retinex Images		
		APCER	BPCER	ACER	APCER	BPCER	ACER	APCER	BPCER	ACER
(**a**)										
**Test-known dataset**	Using Inner Iris Region	0.103	0.099	0.101	0.103	0.000	0.051	0.000	0.000	0.000
	Using Outer Iris Region	0.000	0.050	0.025	0.000	0.100	0.050	0.000	0.000	0.000
	Using Entire Iris Region	0.000	0.050	0.025	0.000	0.100	0.050	0.000	0.148	0.074
**Test-unknown dataset**	Using Inner Iris Region	0.170	0.278	0.224	1.021	1.482	1.251	2.128	0.092	1.110
	Using Outer Iris Region	5.617	0.046	2.832	1.830	3.750	2.790	15.106	0.694	7.900
	Using Entire Iris Region	0.298	0.324	0.311	0.894	0.556	0.725	0.638	0.602	0.620
(**b**)										
**Test-known dataset**	Using Inner Iris Region	0.103	0.198	0.151	0.103	0.000	0.051	0.000	0.050	0.025
	Using Outer Iris Region	0.000	0.000	0.000	0.000	0.010	0.050	0.000	0.000	0.000
	Using Entire Iris Region	0.000	0.000	0.000	0.000	0.000	0.000	0.000	0.000	0.000
	Using Feature Level Fusion Approach	0.000	0.000	0.000	0.000	0.000	0.000	0.000	0.000	0.000
	Using Score Level Fusion Approach	0.000	0.000	0.000	0.000	0.000	0.000	0.000	0.000	0.000
**Test-unknown dataset**	Using Inner Iris Region	0.213	0.324	0.268	4.596	2.130	3.363	0.085	0.509	0.297
	Using Outer Iris Region	0.638	0.787	0.713	0.383	4.444	2.414	2.383	4.259	3.321
	Using Entire Iris Region	0.809	0.370	0.589	0.809	0.833	0.821	0.681	0.139	0.410
	Using Feature Level Fusion Approach	0.213	0.093	0.153	0.383	0.278	0.330	0.170	0.000	0.085
	Using Score Level Fusion Approach	0.128	0.046	0.087	0.213	0.232	0.222	0.000	0.046	0.023

**Table 6:** Description of training and testing datasets with the NDCLD-2015 dataset using LivDet-Iris-2017 division method.

Dataset	Training Dataset			Testing Dataset					
	Real Image	Attack Image	Total	Test-Known Dataset			Test-Unknown Dataset		
				Real Image	Attack Image	Total	Real Image	Attack Image	Total
Original NDCLD-2015 dataset	600	600	1200	900	900	1800	900	900	1800
Augmented dataset	29,400 (600 × 49)	29,400 (600 × 49)	58,800	900	900	1800	900	900	1800

**Table 7:** (a) Detection errors (APCER, BPCER, and ACER) of iPAD based on CNN method for classification using NDCLD-2015 dataset with LivDet-Iris-2017 division method and three kinds of input image (unit: %); (b) Detection errors (APCER, BPCER, and ACER) of iPAD based on SVM method for classification using NDCLD-2015 dataset with LivDet-Iris-2017 division method and three kinds of input image (unit: %).

Test Dataset	Approach	Using Three-Channel Gray Images			Using Three-Channel Retinex Images			Using Three-Channel Fusion of Gray and Retinex Images		
		APCER	BPCER	ACER	APCER	BPCER	ACER	APCER	BPCER	ACER
(**a**)										
Test-known dataset	Using Inner Iris Region	0.056	0.389	0.222	0.167	0.333	0.250	0.167	0.278	0.222
	Using Outer Iris Region	0.000	0.278	0.139	0.056	0.111	0.083	0.000	0.222	0.111
	Using Entire Iris Region	0.000	0.278	0.139	0.000	0.167	0.083	0.056	0.056	0.056
Test-unknown dataset	Using Inner Iris Region	1.278	11.889	6.583	0.444	11.722	6.083	0.333	13.278	6.806
	Using Outer Iris Region	0.056	32.222	16.139	0.278	24.944	12.611	0.222	23.889	12.056
	Using Entire Iris Region	0.389	11.722	6.056	0.222	10.556	5.389	0.222	13.611	6.917
(**b**)										
Test-known dataset	Using Inner Iris Region	0.167	0.111	0.139	0.056	0.389	0.222	0.167	0.111	0.139
	Using Outer Iris Region	0.000	0.278	0.139	0.222	0.000	0.111	0.000	0.167	0.083
	Using Entire Iris Region	0.000	0.278	0.139	0.111	0.000	0.056	0.000	0.111	0.056
	Using Feature Level Fusion Approach	0.000	0.000	0.000	0.000	0.000	0.000	0.000	0.000	0.000
	Using Score Level Fusion Approach	0.000	0.000	0.000	0.000	0.000	0.000	0.000	0.000	0.000
Test-unknown dataset	Using Inner Iris Region	2.167	8.556	5.361	2.278	3.500	2.889	2.722	3.278	3.000
	Using Outer Iris Region	3.611	10.389	7.000	5.167	5.500	5.333	5.611	7.667	6.639
	Using Entire Iris Region	1.333	2.389	1.861	1.556	2.833	2.194	1.389	2.111	1.750
	Using Feature Level Fusion Approach	0.778	2.667	1.722	0.333	0.889	0.611	0.333	0.833	0.583
	Using Score Level Fusion Approach	1.722	1.833	1.778	0.944	0.833	0.889	0.556	1.000	0.778

**Table 8:** (a) Detection errors (APCER, BPCER, and ACER) of iPAD based on CNN method for classification using NDCLD-2015 dataset with our first division method and three kinds of input images (unit: %); (b) Detection errors (APCER, BPCER, and ACER) of iPAD based on SVM method for classification using NDCLD-2015 dataset with our first division method and three kinds of input images (unit: %).

Test Dataset	Approach	Using Three-Channel Gray Images			Using Three-Channel Retinex Images			Using Three-Channel Fusion of Gray and Retinex Images		
		APCER	BPCER	ACER	APCER	BPCER	ACER	APCER	BPCER	ACER
(**a**)										
Test-known Dataset	Using Inner Iris Region	0.389	0.056	0.222	0.111	0.389	0.250	0.278	0.389	0.333
	Using Outer Iris Region	0.000	0.167	0.083	0.000	0.056	0.028	0.000	0.056	0.028
	Using Entire Iris Region	0.000	0.056	0.028	0.000	0.000	0.000	0.000	0.000	0.000
Test-Unknown Dataset	Using Inner Iris Region	1.278	9.778	5.528	0.389	10.778	5.583	0.889	10.667	5.778
	Using Outer Iris Region	0.111	36.611	18.361	0.111	24.944	12.528	0.278	31.389	15.833
	Using Entire Iris Region	0.111	24.667	12.389	0.278	19.444	9.861	0.556	12.944	6.750
(**b**)										
Test-known Dataset	Using Inner Iris Region	0.111	0.444	0.278	0.000	0.556	0.028	0.222	0.278	0.250
	Using Outer Iris Region	0.000	0.167	0.083	0.000	0.000	0.000	0.056	0.000	0.028
	Using Entire Iris Region	0.000	0.000	0.000	0.000	0.056	0.028	0.000	0.000	0.000
	Using Feature Level Fusion Approach	0.000	0.000	0.000	0.000	0.000	0.000	0.000	0.000	0.000
	Using Score Level Fusion Approach	0.000	0.000	0.000	0.000	0.000	0.000	0.000	0.000	0.000
Test-Unknown Dataset	Using Inner Iris Region	3.167	4.944	4.056	2.667	2.833	2.750	2.278	3.889	3.083
	Using Outer Iris Region	2.778	14.000	8.389	2.444	7.333	4.889	3.833	7.556	5.694
	Using Entire Iris Region	1.944	3.389	2.667	2.000	4.333	3.167	1.333	2.278	1.806
	Using Feature Level Fusion Approach	1.222	1.778	1.500	0.389	0.611	0.500	1.056	0.833	0.944
	Using Score Level Fusion Approach	1.556	2.167	1.861	1.167	0.833	1.000	0.722	0.778	0.750

**Table 9:** Description of training and testing datasets of NDCLD-2015 dataset using our second division method.

Dataset	Training Dataset			Testing Dataset		
	Real Image	Attack Image	Total	Real Image	Attack Image	Total
Original entire NDCLD-2015 (1st Fold)	2340	1068	3408	2535	1357	3892
Augmented dataset (1st Fold)	28,080 (2340 × 12)	26,700 (1068 × 25)	54,780	2535	1357	3892
Original entire NDCLD-2015 (2nd Fold)	2535	1357	3892	2340	1068	3408
Augmented dataset (2nd Fold)	30,420 (2535 × 12)	33,925 (1357 × 25)	64,345	2340	1068	3408

**Table 10:** (a) Detection errors (APCER, BPCER, and ACER) of iPAD based on CNN method for classification using NDCLD-2015 dataset with our second division method and three kinds of input images (unit: %); (b) Detection errors (APCER, BPCER, and ACER) of iPAD method based on SVM method for classification using NDCLD-2015 dataset with our second division method and three kinds of input images (unit: %).

Approach	Using Three-Channel Gray Images			Using Three-Channel Retinex Images			Using Three-Channel Fusion of Gray and Retinex Images		
	APCER	BPCER	ACER	APCER	BPCER	ACER	APCER	BPCER	ACER
(**a**)									
Using Inner Iris Region	4.088	31.212	17.650	3.322	35.895	19.608	3.831	34.090	18.961
Using Outer Iris Region	1.851	3.502	2.676	1.921	2.766	2.344	1.767	3.461	2.614
Using Entire Iris Region	1.606	6.120	3.863	1.501	7.845	4.673	1.522	4.418	2.970
(**b**)									
Using Inner Iris Region	6.581	13.810	10.195	6.003	25.649	15.826	5.360	19.749	12.555
Using Outer Iris Region	2.581	1.666	2.123	2.175	0.883	1.529	2.180	1.706	1.943
Using Entire Iris Region	1.907	1.204	1.555	1.898	1.646	1.772	2.079	0.596	1.337
Using Feature Level Fusion Approach	1.481	0.823	1.152	1.777	0.140	0.959	1.649	0.281	0.965
Using Score Level Fusion Approach	1.731	0.599	1.165	1.884	0.094	0.989	1.800	0.214	1.007

**Table 11:** Description of training and testing datasets of fusion of Warsaw-2017 and NDCLD-2015 datasets.

Training Dataset			Testing Dataset					
Images from Warsaw-2017 Dataset	Images from NDCLD-2015 Dataset	Total	Test-Known Dataset			Test-Unknown Dataset		
			Images from Warsaw-2017 Dataset	Images from NDCLD-2015 Dataset	Total	Images from Warsaw-2017 Dataset	Images from NDCLD-2015 Dataset	Total
51,681	58,800	110,481	2990	1800	4790	4510	1800	6310

**Table 12:** (a) Detection errors (APCER, BPCER, and ACER) of iPAD based on CNN method for classification using fusion of Warsaw-2017 and NDCLD-2015 datasets and three kinds of input images (unit: %); (b) Detection errors (APCER, BPCER, and ACER) of iPAD based on SVM method for classification using fusion of Warsaw-2017 and NDCLD-2015 datasets and three kinds of input images (unit: %).

Test Dataset	Approach	Using Three-Channel Gray Images			Using Three-Channel Retinex Images			Using Three-Channel Fusion of Gray and Retinex Images		
		APCER	BPCER	ACER	APCER	BPCER	ACER	APCER	BPCER	ACER
(**a**)										
Test-known Dataset	Using Inner Iris Region	0.160	0.034	0.097	0.053	0.206	0.130	0.000	0.171	0.085
	Using Outer Iris Region	0.053	0.034	0.044	0.053	0.069	0.061	0.053	0.034	0.044
	Using Entire Iris Region	0.000	0.034	0.017	0.107	0.034	0.071	0.053	0.034	0.044
Test-Unknown Dataset	Using Inner Iris Region	0.585	4.020	2.302	2.062	4.575	3.318	1.292	4.412	2.852
	Using Outer Iris Region	3.692	14.183	8.934	3.292	10.458	6.875	5.108	11.765	8.436
	Using Entire Iris Region	0.923	2.386	1.654	0.800	3.726	2.263	0.431	5.621	3.026
(**b**)										
Test-known Dataset	Using Inner Iris Region	0.053	0.034	0.044	0.267	0.343	0.305	0.000	0.172	0.086
	Using Outer Iris Region	0.000	0.069	0.034	0.053	0.000	0.027	0.053	0.000	0.027
	Using Entire Iris Region	0.000	0.000	0.000	0.000	0.000	0.000	0.000	0.000	0.000
	Using Feature Level Fusion Approach	0.000	0.000	0.000	0.000	0.000	0.000	0.000	0.000	0.000
	Using Score Level Fusion Approach	0.000	0.000	0.000	0.000	0.000	0.000	0.000	0.000	0.000
Test-Unknown Dataset	Using Inner Iris Region	0.339	4.935	2.637	3.877	3.595	3.736	2.339	3.105	2.722
	Using Outer Iris Region	4.246	9.510	6.878	4.246	7.353	5.800	4.831	7.811	6.321
	Using Entire Iris Region	1.662	1.536	1.599	2.154	1.144	1.649	1.815	2.222	2.019
	Using Feature Level Fusion Approach	1.231	1.438	1.334	1.200	1.111	1.156	0.862	0.556	0.709
	Using Score Level Fusion Approach	0.400	2.386	1.393	1.015	2.712	1.864	1.354	2.418	1.886

**Table 13:** The processing time of our proposed iPAD method (unit: ms).

Pupil and Iris Boundary Detection	Inner and Outer Region Image Extraction	Retinex Filtering	Deep Feature Extraction	Feature Selection by PCA	Classification by SVM	Total
22.500	3.776	0.011	58.615	0.0001	0.00002	84.90212

**Table 14:** Comparison of detection errors (ACER) between proposed method and previous methods using Warsaw-2017 and NDCLD-2015 datasets (unit: %).

Method	Warsaw-2017 Dataset			NDCLD-2015 Dataset		
	APCER	BPCER	ACER	APCER	BPCER	ACER
CASIA method [34]	3.40	8.60	6.00	11.33	7.56	9.45
Anon1 method [34]	6.11	5.51	5.81	7.78	0.28	4.03
UNINA method [34]	0.05	14.77	7.41	25.44	0.33	12.89
CNN-based method [25,38]	0.198	0.327	0.263	1.250	5.945	3.598
MLBP-based method [57]	0.154	0.285	0.224	4.056	7.806	5.931
Feature Level Fusion of CNN and MLBP Features [25]	0.154	0.131	0.142	1.167	3.028	2.098
Score Level Fusion of CNN and MLBP Features [25]	0.000	0.032	0.016	1.389	4.500	2.945
Our proposed method	0.000	0.032	0.016	0.167	0.417	0.292
